# Language may indeed influence thought

**DOI:** 10.3389/fpsyg.2015.01631

**Published:** 2015-10-31

**Authors:** Jordan Zlatev, Johan Blomberg

**Affiliations:** ^1^Centre for Languages and Literature, Lund UniversityLund, Sweden; ^2^Centre for Languages and Literature/Department of Philosophy, Lund UniversityLund, Sweden; ^3^Institut für Sprache und Kommunikation, Technische Universität BerlinBerlin, Germany

**Keywords:** consciousness, culture, discourse, language, relativity, thought, Whorf

## Abstract

We discuss four interconnected issues that we believe have hindered investigations into how language may affect thinking. These have had a tendency to reappear in the debate concerning linguistic relativity over the past decades, despite numerous empirical findings. The first is the claim that it is impossible to disentangle language from thought, making the question concerning “influence” pointless. The second is the argument that it is impossible to disentangle language from culture in general, and from social interaction in particular, so it is impossible to attribute any differences in the thought patterns of the members of different cultures to language *per se*. The third issue is the objection that methodological and empirical problems defeat all but the most trivial version of the thesis of linguistic influence: that language gives new factual information. The fourth is the assumption that since language can potentially influence thought from “not at all” to “completely,” the possible forms of linguistic influence can be placed on a cline, and competing theories can be seen as debating the actual position on this cline. We analyze these claims and show that the first three do not constitute in-principle objections against the validity of the project of investigating linguistic influence on thought, and that the last one is not the best way to frame the empirical challenges at hand. While we do not argue for any specific theory or mechanism for linguistic influence on thought, our discussion and the reviewed literature show that such influence is clearly possible, and hence in need of further investigations.

## Introduction

The two related questions *if* and *how* language affects the mind go back to the dawn of contemplative thought. Since thought and language are intimately connected, some form of close relationship between the two has often been assumed. The recurrent debate, with oscillating tendencies, has been whether it is mostly thought that influences language, or vice versa (Zlatev, 2008a). The thesis that language has a non-negligible effect on thinking, combined with the claim that languages are non-trivially different, has been generally known as “the Sapir–Whorf hypothesis.” This is a rather misleading label, introduced by Carroll (1956) in the preface to the well-known collection of papers by Benjamin Lee Whorf *Language, thought and reality*. In fact, the original idea did not amount to an empirical hypothesis, but to what we would today call a “research program,” and its main promotor was Whorf. With 60-years long hindsight, we can now observe that after a prolonged period of scientific mistrust, what Whorf (1956, p. 213) dubbed *the principle of linguistic relativity* appears to find a substantial degree of support in interdisciplinary research from the past two decades (Lucy, 1992, 1997; Pederson, 1995; Gumperz and Levinson, 1996; Slobin, 1996; Boroditsky, 2001; Gentner and Goldin-Meadow, 2003; Levinson, 2003; Casasanto et al., 2004; Majid et al., 2004; Casasanto, 2008; Casasanto and Boroditsky, 2008; Boroditsky and Gaby, 2010; Wolff and Holmes, 2011; Lupyan, 2012).

At the same time, the thesis that language influences thought, in one or more possible ways, especially when combined with the thesis of linguistic relativity, continues to be highly controversial, and every now and then provokes sweeping criticisms, describing the enterprise as fatally flawed (Pinker, 1994; McWhorter, 2014). On the other hand, some proponents of the thesis have also been relatively one-sided (Durst-Andersen, 2011). Perhaps it is as stated by Ellis (1993, p. 55): “[T]he Whorf hypothesis seems to bring out the worst in those who discuss it.”

In this article, we wish to take a few steps back, and consider the following objections that have been leveled at the project. First, some have proposed that the question of language influencing thought is conceptually unsound: since the two cannot even be distinguished, thought cannot exist independently of language. A second objection is that it is impossible to disentangle language from culture in general, and from social interaction in particular, so it is impossible to attribute differences in the thought patterns of the members of different cultural communities to the structures of language. A third critique states that the strong thesis of linguistic influence is methodologically circular, or else false, while the weak thesis is trivial. A fourth issue is not so much an objection as something that has been presented in the way of a practical solution to the dilemma: since language can potentially influence thought from “not at all” to “completely,” theoretical proposals can be arranged on a cline from “weak” to “strong,” and the only issue is to determine the place of linguistic influence on the cline, presumably toward the weak end.

We examine each one of these issues in turn. To anticipate, concerning the first three objections, we propose that the force of the critique has been overstated and the conceptual problems can be avoided. With respect to the final point, we argue that at least some “language influence” theories differ not quantitatively but qualitatively, according to two independent dimensions. Our aim is thus to show that much of the dismissive critique against language-thought influence and linguistic relativity is unsatisfactory, and thereby to pave the way for further research. While we often refer to relevant empirical findings, our aim is not primarily empirical – to answer *how* exactly language influences thought – but to clarify the semiotic space surrounding the debate. The outcome of this clarification is (minimally) the conclusion that it is fully possible for language to influence thought, and that it remains to determine the ways in which this possibility is actualized in practice (Wolff and Holmes, 2011). Such cross-fertilization of conceptual and empirical concerns is characteristic of the new field of *cognitive semiotics* (Zlatev, 2012) which the present approach instantiates.

## Disentangling Language from Thought

A classical objection against the possibility of cogently posing the question of linguistic influence on thought is to reject the proposition that the latter could even exist in the absence of language. Philosophers, at least since Humboldt (who wrote: “…the idea is born, becomes an object and returns, perceived anew as such, into the subjective mind. For this, language is inevitable,” quoted and translated by Zinken, 2008, fn. 10), have often been inclined to such a radical position, implying that without language we would be thought-less, or even mindless. While this view still has its champions among philosophers (Dennett, 1991; Macphail, 1998), it is harder to find it represented in psychology or the language sciences. Still, some researchers following Humberto Maturana (e.g., Maturana, 1988), who placed an especially heavy emphasis on the role of language (or languaging) for the “construction of reality,” appear to accept a version of this view:

An existing impasse in the study of this relationship (i.e., between language and mind) cannot be overcome as long as the problem itself is not reformulated to rid it of the intrinsically dualistic assumption that there is, in fact, a phenomenon called ‘language’ that is ontologically independent of the phenomenon called ‘mind.’ […] mind cannot be understood without and outside of language.

(Kravchenko, 2011, p. 355)

It is quite possible to agree with such claims in some respects, e.g., that treating language and thought as fundamentally different “modules” or “representations” is mistaken (Lupyan, 2012), but nevertheless maintain that language and thought should not be equated, since doing so would short-circuit the crucial question concerning their interrelation (Vygotsky, 1962).

A convenient definition of language, adopted in some of our earlier work is that of *a predominantly conventional semiotic system for communication and thought* (Zlatev, 2007, 2008b). This comprises the point that languages are essentially “socially shared symbolic systems” (Nelson and Shaw, 2002), which have evolved over millennia and develop in children over years, to serve two main functions: sharing experiences and enhancing cognition. Indeed, this definition implies that thought is not impossible without language and that it is possible to treat the two phenomena as distinct, e.g., “Language invades our thinking because languages are good to think with” (Bowerman and Levinson, 2001, p. 584). By “thought,” we mean essentially *mediated cognition*. This corresponds approximately to what are sometimes called “higher cognitive processes,” in which the mind is not fully immersed in the practical concerns of the here-and-now, but rather employs various structures and processes of conscious awareness such as mental imagery, episodic memories or explicit anticipations to focus on intentional objects that are not perceptually present. We believe that this corresponds fairly well to the folk-psychological concept of “thought” and “thinking.” It is worthwhile distinguishing this, at least analytically, from non-mediated forms of cognition, including (conscious and non-conscious) processes of perception, movement, procedural memory and implicit anticipation. We propose that the issue of “linguistic influence on thought” can be circumscribed in this way. This does not exclude the possibility that language may in some cases even “modulate” perception (Lupyan, 2012), as the attested presence of such modulation – in nearly all cases found to be transitory and context-dependent – can also be interpreted as an instance of linguistic mediation.

Given these explications of the key concepts, what is the evidence that it is language alone that can give rise to thought, or in other words: serve as the “sole mediator” of cognition? Phenomenological analysis (e.g., Merleau-Ponty, 1962/1945; Husserl, 1989/1952) and psychological research all show that mediated cognition is possible without language. For example, monkeys are capable of making *decisions* on the basis of *judgments* of whether a given stimulus is familiar or not, which is difficult to explain without episodic memory (Griffin and Speck, 2004). Chimpanzees and orangutans are apparently capable of *planning* for the (near) future (Osvath and Osvath, 2008), and at least chimpanzees and bonobos display behaviors such as consolation and tactical deception which require one to place oneself “in the shoes” of someone else, known as *cognitive empathy* (Preston and de Waal, 2002). Of course, there are forms of thinking that are indisputably linguistically mediated: internal speech, complex planning, and an autobiographic self-concept (Nelson, 1996). Few would doubt that language plays a constitutive role in such “linguistic thought,” though many questions remain concerning the extent to which this is so, and by which “mechanisms” this is realized (Bowerman and Levinson, 2001; Casasanto, 2008; Wolff and Holmes, 2011). The point is that not all instances of thought, and even less so of cognition in general, are co-extensive with language. Thus, the question of linguistic influence on thought can be formulated fairly simply: *to what extent and in which ways does language mediate cognition?*

A counter-claim could be that even if thought and language can be in principle (ontologically) distinguished, this is not possible *methodologically* – for “languaging” creatures such as us. This issue presents itself clearly in empirical research on linguistic relativity: as with Einstein’s principle of relativity, some form of stable “reality” is presupposed to be able to establish the differences between “measurements” or perspectives in the first place. This reality need not, as the invariance of light in Einstein’s theory, be understood as something that is strictly mind-independent, but rather as the world of perception (Merleau-Ponty, 1962/1945). Many readers of the supposed relativist Whorf are surprised to find multiple references to such a universal level of experience.

To compare ways in which different languages differently ‘segment’ the same situation of experience, it is desirable to analyze or ‘segment’ the experience first in a way independent of any language or linguistic stock, a way which will be the same for all observers

(Whorf, 1956, p. 162).

In describing differences between [languages] … we must have a way of describing phenomena by non-linguistic standards and by terms that refer to experience as it must be to all human beings, irrespective of their languages or philosophies

(Whorf and Trager, 1938, p. 6).

Even if other passages of Whorf’s writings can be read as proposing that thought is fully dependent on language (Brown, 1976), quotations such as these clearly show that Whorf accepted a pre-linguistic mode of representation that was yet unaffected by language, and hence the need to compare languages with respect to the degrees to which they departed from such experience. Indeed, as the quotations above imply, Whorf even regarded this as a methodological necessity. This position is accepted in all current empirical research in linguistic relativity, such as the active field of motion event typology (Talmy, 2000), where it is investigated whether cross-linguistic differences in motion expressions correlate with non-linguistic categorizations (e.g., Slobin, 2003). Such studies presuppose prior analyses of the domain itself, i.e., analyses that necessitate the possibility to classify experience “independent of any language or linguistic stock.” In previous work, we have proposed exactly such an analysis of motion on the basis of three binary parameters (TRANSLOCATIVE, BOUNDED, CAUSED), distinguishing between eight kinds of motion situations (Zlatev et al., 2010). This provided a better conceptual basis for describing semantic differences between languages in the expression of motion (Blomberg, 2014) than the original Talmian framework. Such an analysis is a necessary precondition for asking Whorfian questions.

To summarize, defining language and thought in a way that is both true to the phenomena, and allowing them to be both distinguished and co-related, is a first prerequisite for further investigations into their relationship. Occasional claims that such distinction is ontologically or methodologically impossible seem to derive from strong theoretical biases rather than from conceptual necessity or empirical evidence.

## Disassociating Language and Cultural Context

Somewhat similarly to the critique from the previous section Björk (2008) argues that current studies in linguistic relativity, often referred to as “neo-Whorfian” (cf. McWhorter, 2014) adopt a simplified and static view of language:

The neo-Whorfian studies investigate the role of linguistic diversity in the language-and-thought relation, and language is thus explored primarily as ‘particular languages,’ such as English, Tzeltal, Dutch, or Yucatec Maya. The particular languages are viewed as demarcated, cognitively represented systems, in which linguistic meaning is inherent. That is, linguistic meaning is given by the system, prior to any particular situation of language use. The term ‘language,’ that sometimes comes into the discussion about relativity as opposed to ‘languages,’ seems to refer to general aspects of having ‘a language,’ a code. When communication is mentioned, this too seems to be a general aspect of using ‘a language.’

(Björk, 2008, pp. 125–126)

Of course, there is more to language than using a particular ‘code’: actual, situated language use, which is also tightly intertwined with socio-cultural practices. For example, a study of linguistic effects on spatial cognition would be simplistic if it only considered “spatial expressions” like prepositions. These should rather be seen as elements in social practices, or “language games” (Wittgenstein, 1953), inseparable from the activities in which they participate in, like asking for directions and specifying the location of objects, events, places, and people. In other words, language needs to be understood as *socio-culturally situated*: “Linguistic meaning is inextricable from the social practices (language games), in which language is used. The mastery of a language is embedded in, and in important ways formative of, one’s cultural background” (Zlatev, 1997, p. 5). Consequently, it is only actual linguistic practices that can have an effect on thought. To claim that linguistic *structures* – as a distinct and separate “variable” – can function as causes of cognitive differences in the speakers of different languages is to evoke abstract, and ontologically suspicious, entities as causes (Berthele, 2013).

As before, we can in part agree with such a critique, but believe that it both exaggerates the problem, and underestimates the methodological sophistication of neo-Whorfian research, where factors such as usage frequency are taken into account (Slobin, 1996; Casasanto, 2008). Conceptually, the notion of language should indeed include, and possibly even privilege situationally and culturally embedded discourse. But this does not mean that the ontology of language should be restricted to such discourse, and thereby exclude “particular languages,” such as English, Tzeltal, Dutch, or the general notion of having a language, associated with particular universal properties (such as *displaced reference* and *predication*). These three aspects: situated discourse, particular language and language in general actually appear as distinct levels of language in the meta-linguistic framework of Coseriu (1985), as shown in his matrix of levels and perspectives, displayed in **Table 1**. This explicitly pluralistic and non-reductionist linguistic ontology (cf. Zlatev, 2011) not only acknowledges the existence of universal, historical, and situated levels of language (vertically), but also of different perspectives on each of these (horizontally): language as creative activity, as competence and as product. All these are to some degree independent, but complementary and interacting aspects of language. In line with Björk (2008), we can agree that the most “real” or actual aspect of language is that of discourse, since it is both most “alive,” unfolding in the communication between speakers and hearers, and most contextualized. At the same time, discourse will be constrained by the grammatical and semantic norms of the particular language, as well as of potentially universal aspects of pragmatics, such as *the cooperative principle* (Grice, 1975). While the linguistic norms of a language community do not determine actual speech, and hence the thought processes related to it, the “historical” level clearly influences that of discourse, in a way that is analogous to the ways social norms influence social behavior (Itkonen, 2008).

**Table 1:** Coseriu’s matrix; adapted from Coseriu (1985; see also Zlatev, 2011), highlighting *Discourse* as the privileged, but non-exclusive aspect of language.

	Perspectives		
Levels	Activity	Competence	Product
Universal	Speaking in general	Encyclopedic and logical	Totality of utterances
Historical	Speaking a particular language	Linguistic	“Lexicon and grammar”
Situated	*Discourse*	Communicative	Text

The discussion so far concerned the relations between language-as-system and language-as-discourse, showing that while the two are closely related, the system level is neither an epiphenomenon, nor a figment of the imagination of (structural) linguists, and hence has the potential to be “causally efficacious.” However, one may grant this, but still deny that the system of lexical and grammatical norms can be dissociated from other aspects of culture, such as shared beliefs and attitudes. Thus, to the extent that there are differences in thought, these should be attributed to cultures rather than languages (cf. McWhorter, 2014). In fact, Whorf and his predecessors Boas and Sapir, always considered the possibility of cultural beliefs and practices interacting with “grammatical patterns as interpretations of experience” (Whorf, 1956, p. 137) in a reciprocal manner. It has, however, been more difficult to provide evidence for a direct causal link from such beliefs to any aspect of “habitual thought” that could be empirically attested. Everett’s (2005) proposal that the high value that the Pirahã attach to “direct experience” is the main reason for their language lacking numerals and many aspects of grammatical complexity such as hierarchical structure, is a case to the point: while not lacking plausibility, the claim has remained strongly controversial and difficult to validate. A stronger case can be made that it is the “habitual patterns” of language – possibly reflecting some particular aspect of the respective culture – that exercise such effects. As Levinson (2005, p. 638) writes:

Everett […] prefers an account in terms of the causal efficacy of culture, but no one interested in language diversity would make a simple dichotomy between language and culture: a language of course is a crucial part of a culture and is adapted to the rest of it. […] The question neo-Whorfians are interested in is how culture gets into the head, so to speak, and here language appears to play a crucial role: it is learnt far earlier than most aspects of the culture, is the most highly practiced set of cultural skills, and is a representational system that is at once public and private, cultural, and mental.

Methodologically, studies have been designed so as to attempt to tease apart the respective roles of language and other aspects of culture, for example by including speakers from languages where certain particular linguistic structures are similar, while there are many other cultural differences, e.g., Yucatec Maya and Japanese (Lucy, 1992). Indeed, in this study the participants of the two groups behaved similarly with respect to object categorization, and differently from, e.g., speakers of English, and this could be plausibly attributed to the extensive use of nominal classifiers in both Yucatec Maya and Japanese.

Conversely, one may test speakers from populations that are very similar culturally, and even linguistically – apart from one particularly relevant variable. This was the case in Pederson’s (1995) study comparing speakers of Tamil who preferentially used a “relative” reference frame for locating objects in space, with terms corresponding English *left-right-front-back*, with another group of Tamil speakers who were familiar with this usage, but preferred to use an “absolute” reference frame, with terms corresponding to *north-south-east-west*. In other words, what one group would tend to phrase as ‘the glass is to the left of the plate,’ would be preferentially expressed in terms of cardinal directions, e.g., ‘the glass is to the west of the plate,’ by the other. In experiments of the type that have since then been used for a number of languages (Levinson, 2003), it was shown that the two groups tended to solve non-linguistic spatial tasks in ways that corresponded to their linguistic preferences. These results are important since as Pederson (1995, p. 40) writes, “this difference in habitual language use is not deeply rooted in the grammatical system,” i.e., it was not a matter of obligatory or “abstract” properties of two substantially different languages, but was rather a matter of preferences of two very closely related dialects. Still these were sufficient to give rise to differences in solving (apparently) non-linguistic tasks.

Finally, the fact that there is a debate concerning the respective causal roles of language structures and non-linguistic cultural patterns is indicative enough that the distinction is not only conceptually possible, but also empirically useful. Ultimately, empirical evidence should be able to resolve some particular debates on this matter. For example, Ji et al. (2005) reported differences in styles of visual attention (“analytic” vs. “holistic”) between East-Asian and American participants, and attributed these to non-linguistic cultural differences: individualist vs. collectivist values, respectively. Durst-Andersen (2011) disagrees, and rather places languages as diverse as Chinese, Russian, and Spanish in the (super)type of “reality-oriented” languages, on the basis of common structural features such as grammatical aspect. This implies that Russian and Spanish speakers should behave like the Chinese, rather than the North Americans in visual attention tasks. To the extent that this prediction holds, the Whorfian interpretation would be supported; if not, the proposal for some degree of “cultural relativity” would retain its credibility. Finally, one may note that what makes Nisbett’s thesis of cultural relativity more testable than Everett’s, mentioned earlier, is precisely that it does not concern a single culture but many different ones, according to a hypothetical typology. It is this that allows formulating contrastive predictions.

## “Interesting” and “Trivial” Kinds of Language Influence?

In an influential review article, Bloom and Keil (2001) made the distinction between two kinds of claims/theories of linguistic influence on thought, referring to the first as “interesting” and the second as “trivial”:

[W]e do want to insist on the distinction between the interesting claim that language induces theory change because of *linguistic structure* (e.g., the particular words it has) vs. the trivial claim that language induces theory change because of the information it conveys. There is a big difference, after all, between arguing that children’s developing theory of, say, the social world is shaped by the specific lexical division that their languages make (interesting) vs. arguing that children’s developing theory of the social world is shaped by what they hear people talking about (trivial).

(Bloom and Keil, 2001, p. 362, original emphasis)

This passage merits some explanation. The authors here assume a “theoretical” perspective on cognitive development, according to which we build up (implicit) theories about the world, including “theories” about other human beings and ourselves (Gopnik and Meltzoff, 1997). Hence, any act of cognition that gives us new knowledge can be seen as “theory change.” Now, it can be reasonably objected that cognition, and even thought (in the sense of mediated cognition, see Disentangling Language from Thought) involves processes such as episodic memory, foresight and imagery that are very hard-pressed into the frame of “theorizing.” But we may ignore this, since the distinction that Bloom and Keil (2001) evoke should remain even if we substitute “induces theory change” with “influences thought” in the quotation above.

So what is meant by “linguistic structure” and why should its possible influence on thought be “interesting”? At first look, one may think that this refers to a distinction made already by Whorf (1956): the more limited effects of lexical items, like calling a barrel with dangerous fumes *empty*, and the much more pervasive effect of “grammatical patterns” (i.e., morphology and syntax), which are used ubiquitously, and under less conscious control. However, Bloom and Keil (2001) specifically refer to “the particular words” a language has in exemplifying what they mean by structure, which is indeed consistent with the rejection of “a simple dichotomy between lexical and grammatical elements” (Croft, 2003, p. 226) in most contemporary linguistics.

In fact, the distinction that the authors are aiming at corresponds to the distinction between the *historical* (“structure”) and the *situated* levels (“talk”) of language discussed in the previous section (see **Table 1**). However, while we argued that *discourse*, or actual situated language use is what has the potential to influence thought, Bloom and Keil (2001) assume that only system-level linguistic differences are worthy of being considered as (interesting) causes of cognitive differences. On the face of it, this is puzzling, since linguistic structures are always realized in discourse (“talk”), and talk is never un-structured. Why should the effects on children’s cognitive development of “what they hear people talking about” be considered trivial? Apparently, since discourse and the knowledge it yields are so pervasive: nearly everything that we learn without direct perceptual experience is linguistically mediated (and more recently, pictorially mediated as well): dinosaurs, angels, Mount Everest, quarks, genes, etc. For example, the word *quark* denotes a certain class of objects hypothesized by modern physics. By means of the informational content of the term we delineate, if not establish, the concept of the basic constituent of matter. Still, Bloom and Keil (2001) discount such cognitive effects, since words like *quark* apparently do not constitute a systematic aspect of language.

However, the distinction “information vs. structure” is problematic. As is well-known since at least de Saussure (1916), the meaning of words in not exhausted by their referential (“informational”) content, but also involves the web of relations to other words. To take the previous example, the words *quark*, *basic*, *constituent* and *matter* can be seen as systematically interrelated: their meanings are to some degree inter-defined, as well as in relation to the “language game” of modern science that they participate in. To take another example: is it not a structural aspect of English that *dinosaurs* are (considered to be) *reptiles*, while *elephants* are *mammals*, and so are *dolphins*, though the latter were thought for a long time to be *fish* (and still are in many other languages/cultures)? Such structure, as well as that encoded in “grammatical patterns” of language, will of course provide “information” during language learning and everyday use. Thus, the dichotomy between information and structure that Bloom and Keil’s view rests upon cannot be upheld: linguistic information is always structured, and structural distinctions are informative.

Further, if we consider the example of social cognition, used by Bloom and Keil (2001) in the citation above, there is considerable evidence that language contributes strongly to children’s understanding of the concept of belief (and hence of “false beliefs”). Indeed, at least two undeniably structural features of language have been argued to contribute to this: (a) mental predicates such as *think*, *believe*, *know*… and (b) sentential complement constructions such as *say that* (de Villiers and Pyers, 1997; Astington and Jenkins, 1999). On the other hand, others have argued that such features are not the only, and possibly not the primary factors that allow language acquisition to influence social cognition. Tomasello (1999, p. 173) suggests that typical features of linguistic interaction such as disagreements, repairs and explanations constitute (at least) “three kinds of discourse, each of which requires [children] to take the perspective of another person” (Lohmann and Tomasello, 2003). Finally, Hutto (2008) presents a book-length argument that the crucial aspect of language that leads to proficiency in “folk psychology” are all the stories that children are told. In sum, both structural and informational aspects of language are likely to contribute to developing concepts such as wish, intention, reason, belief, and even more so for interrelating them into discursive and holistic complexes such as “folk psychological narrative.” Since the distinction between “talk” and “structure” (and hence of their possible effects on thought) is highly dubious, there is nothing obviously trivial about the influence of the former.

Let us consider the other prong of the dilemma that Bloom and Keil (2001) set up for linguistic relativity (“interesting, but wrong”). They first point out a standard methodological objection: that Whorf and many who have gone in his footsteps use a circular argumentation where linguistic differences are the sole evidence for cognitive differences. In fact, Whorf was aware of this problem, and pointed out the need for future studies to corroborate his conjectures (Whorf, 1956, p. 162). One can say that documenting linguistic diversity is a necessary preliminary step to formulating hypotheses of linguistic influence. We may employ Popper’s (1935) distinction between “context of discovery” and “context of justification,” and regard Whorf as engaged in the first, while modern neo-Whorfians with psychological training clearly aim for the latter:

A full theory of the relation of language diversity to thought necessarily involves at least *three logical components*. It must distinguish between language and thought in some principled way. It must elaborate the actual mechanisms or manner of influence. And it must indicate to what extent other contextual factors affect the operation of these mechanisms.

(Lucy, 1997, p. 306, original emphasis)

Still, Bloom and Keil (2001) find faults even with the studies that follow such a procedure. For example, Lucy’s studies in object categorization on the basis of shape vs. material in speakers of different languages did not show differences in 7-years-old children; differences in spatial reasoning such as those of Pederson (1995) can be due to ecological, rather than linguistic differences; showing that language is essential for numerical reasoning (Dehaene, 1997) may also turn out as trivial: “if the task itself requires that the person use inner speech, for instance, then any effect of language on performance is considerably less interesting” (Bloom and Keil, 2001, p. 358). Thus, the authors reach the conclusion that has been hinted since the onset of their review: “taken together… the available research does not challenge the mainstream view (ibid: 364)” that language is a module quite separate from thinking, or even more plainly: “the language you speak does not affect how you think” (ibid: 351).

We have spent considerable time on one particular paper, albeit as mentioned an influential one, not so much because we disagree with the factual conclusions of the authors, but because we find its style of reasoning quite typical for “mainstream” cognitive science (e.g., Pinker, 1994), where notions of (innate) “modules,” “information processing” and “mental representations” are axiomatic. Since there is no logical possibility for language to influence thought (in any “interesting” way) given such a conceptual apparatus, the strategy is first to split the claim of linguistic influence into “discourse-based” and “structure-based.” The former is then deflated as a truism, while the second is demolished methodologically, or reduced to the trivial variety. Ironically, one could suggest that cognitive scientists like Bloom, Keil, and Pinker are so influenced by the language-based conceptual framework they work with, that their conclusions are (almost) predetermined.

Our main counter-objection to this line of reasoning has been that the distinction between “information” and “structure” corresponds to the distinction between discourse (situated) and language system (historical) in Coseriu’s framework, discussed earlier. Since the two aspects of language presuppose one another, they cannot be opposed as “trivial” vs. “interesting.” Admittedly, different kinds of (possible) linguistic influence on thought need to be distinguished, and some may be more pervasive than others. Thinking of dolphins as mammals might change ways of reasoning (and ethics), but will hardly affect reasoning in other domains. On the other hand, the presence of a linguistic “structure” such as the obligatory grammatical marking of the evidence the speaker has for every proposition (direct experience, inference, hearsay, etc.), a feature of, e.g., Turkish, could turn out to have much more wide-ranging influences. The extent of such influence is what remains to be determined, but to rule it out is clearly premature.

## Different Kinds of Theories of Linguistic Influence

By insisting on a qualitative distinction between “interesting” and “trivial” linguistic influence, Bloom and Keil (2001) were in one way atypical: the so-called Sapir–Whorf hypothesis is commonly divided into a “weak” and a “strong” version, as in the following formulations by Brown (1976, p. 128):

(1) Structural differences between language systems will, in general, be paralleled by non-linguistic cognitive differences, of an unspecified sort, in the native speakers of the two languages.
(2) The structure of anyone’s native language strongly influences or fully determines the world-view he will acquire as he learns the language.

Can one apply such a distinction to the thesis of linguistic influence on thought in general? The adjectives *weak* and *strong* are gradient opposites, entailing the existence of a continuum ranging from approximately zero (“no influence”) to maximum (“complete determinism”). If so, specific theoretical proposals of linguistic influence such as those of Whorf (1956), Vygotsky (1962), Lucy (1992), Levinson (2003), etc. can in principle be arranged on a cline representing “strength of influence.” The main issue would be to establish which proposal corresponds to the actual position on the cline – and if following the reasoning of Bloom and Keil (2001) it should be somewhere very close to the “no influence” end.

We find such a gradient conception of linguistic influence misleading for at least two related reasons. First, at least four types of (possible) linguistic influence – and corresponding theoretical proposals – differ from each other not quantitatively but qualitatively. Second, at least three of these types of influence are not mutually exclusive or incommensurable with one another, and could potentially all be valid. A similar argument has been made in a recent review article (Wolff and Holmes, 2011), but here we follow the distinctions made by Blomberg and Zlatev (2009), where theories of linguistic influence on thought are distinguished according to two parameters. The first parameter is context. Whorf’s (1956) principle of linguistic relativity is, for example, *context-general:* irrespective of the task, context or situation, some particular aspect of language will influence one’s thinking, at least in some particular domains. A *context-specific* type of influence, on the other hand, gives more freedom to thought, and allows a particular task to be solved either without, or if necessary, with the help of language. The second parameter concerns whether features of particular languages affect thinking (*language-specific*) as in the Whorfian tradition, or if the properties of language that influence thought are so general (e.g., prediction, hierarchical structure) that there would be no differences between language communities in the way that language affects thought (*language-general*), as opposed to the difference of having or not having language. These two parameters/dimensions can be combined, yielding four types of linguistic influence, each represented by a number of theories, as shown in **Table 2**.

**Table 2:** Four general kinds of theories of linguistic influence on thought (with example references, discussed in the text), categorized on the basis of the binary parameters: *Context*: general vs. specific and *Language*: specific vs. general.

Language/Context	Specific	General
Specific (relativistic)	Type 1 Whorf, 1956; Lucy, 1992; Levinson, 2003	Type 2 Pederson, 1995; Slobin, 1996; Casasanto, 2008; Lupyan, 2012
General (non-relativistic)	Type 3 Dennett, 1991; Macphail, 1998	Type 4 Vygotsky, 1962; Tomasello, 1999

As stated, it is not our intention to evaluate in detail each of the theories of linguistic influence categorized in **Table 2**. We need, however, say a few words concerning each type for the purpose of justifying our classifications, and to support our claim that not all of these are mutually exclusive. We should also add that these categories are somewhat over-schematic, glossing over distinctions between theories within each type. Still, they serve the purpose of the main point that we are making: that language-upon-thought theories do no fall on a “strong-weak” continuum.

Type 1, classically represented by Whorf (1956), remains viable as long as there is a plausible “mechanism” according to which linguistic classification can affect thinking so pervasively as to be available in any kind of context and situation. Both Lucy (1992) and Levinson (2003) give explanations for how this could take place: through making the respective distinctions encoded in the language from the onset of language acquisition, and thus in the words of Evans (2010, Chap. 8), “training thought” to make the corresponding distinctions. In the terms of the divisions made by Wolff and Holmes (2011), this concerns the roles of language as “spotlight” and “inducer.” Levinson’s findings that speakers of languages using (only) *absolute* frames of spatial reference, also use these frames in thinking, navigating and gesturing, constitute some of the strongest evidence for a language-specific, context-general type of effect.

Type 2, which is similarly language-specific, but also context-specific, may be represented by Slobin’s (1996, p. 76) *thinking for speaking* hypothesis, according to which linguistic structure (see “Interesting” and “Trivial” Kinds of Language Influence?) affects the “thought that is mobilized for speaking.” Slobin does not exclude more general effects, but has focused on what is apparently the most obvious context of linguistic influence: the distinctions that are made while using language. This may be a rock-bottom of linguistic influence, since even well-known opponents of the thesis of linguistic influence seem to accept it: “one’s language does determine how one must conceptualize reality when one has to talk about it” (Pinker, 1989, p. 360). Other theories within this category make more substantive proposals. Pederson’s (1995) study of Tamil speakers who preferentially used either relative or absolute frames of reference (unlike Levinson’s more mono-frame speakers of Guugu Yimithirr), displayed only a strong tendency to solve the spatial task in a manner that corresponded to their preferred linguistic usage. Thus, Pederson (1995, p. 54) concludes that language cannot be used as “obligatory means,” but only optionally: “Under the weaker language as optional means hypothesis, the experimental results suggest a significant, close and variable relationship between language and thought.” Another testified effect that could be grouped here as a “stronger,” but still context-specific type of influence, are the findings that English speakers could be induced after relatively short periods of exposure to think of time in terms of Greek-style CONTAINER metaphors (‘large’ and ‘small’ quantities of time) and thus “override” the conventional LENGTH metaphors of ‘short’ and ‘long’ distances of time used in their native tongue (Casasanto et al., 2004). With respect to Wolff and Holmes (2011) classification, this could be seen as an instance of language as “meddler,” with linguistic representations influencing non-linguistic cognition differently on different occasions, depending on multiple factors that for the sake of simplicity we may call *context*. Lupyan’s (2012) “label-feedback hypothesis,” aiming to account for both the pervasiveness of language-cognition effects and for their fragile character (e.g., they are easily disrupted by verbal interference), would also fall within this category of theories, as shown in the methodological conclusion: “in may be more productive to measure the degree to which performance on *specific tasks* is being modulated by language, modulated differently by different languages, or is truly independent of any experimental manipulations that can be termed linguistic” (ibid: 10).

Turning to the language-general, non-relativist type of linguistic influence, Type 3 represents the possibility that was discussed (and rejected) in Section “Disentangling Language from Thought”: that language more or less “creates” thought, or even consciousness. Perhaps the foremost representative of this position in the current debate is Dennett (1991), with his famous (if rather mysterious) claim that:

Human consciousness is itself a huge complex of memes (or more exactly, meme effects in brains) that can best be understood as the operation of a von Neumannesque virtual machine implemented in the parallel architecture of a brain that was not designed for such activities.

(Dennett, 1991, p. 210)

Macphail (1998) attempts to justify such a claim empirically by considering (and discounting) various evidence for animal consciousness. It is somewhat unclear if this implies returning to the discredited Cartesian view of animals as “mindless automata” and if this also applies to pre-linguistic children. In any case, even if Type 3 is conceptually problematic, ethically detestable and empirically implausible (Griffin and Speck, 2004), it is worth considering as part of the global picture, striking out a (remote) space of possibilities.

Finally, Type 4 is the much more palatable version of linguistic influence often associated with the notion of *linguistic mediation* of Vygotsky (1962, 1978). According to this view, language is analogous to a tool insofar as it enables us to solve a certain task more easily than would have been the case if the same task were approached with non-linguistic thought. Differences between languages may be less relevant (though should not be excluded) than the fact of using, or not using language. For example, Zlatev et al. (2010) found that Swedish and French speakers solved a non-linguistic task involving the categorization of animated motion events in a similar way when they described these events prior to the similarity judgement. This was despite relevant semantic differences between the languages that would have been expected to lead to different similarity judgments, in a Type 2, thinking-for-speaking scenario. Also, Tomasello’s (1999) argument that the “perspectival” nature of linguistic symbols and certain forms of discourse, mentioned in the previous section, play an important role for bringing about the understanding of others as “mental agents” with beliefs, intentions and emotions, can also be regarded as belonging to this class of language-general, context-specific effects on thought.

To repeat, distinguishing types of linguistic influence in the manner proposed here may be too schematic, but it serves the purpose of our particular argument: to show that it is conceptually inaccurate and analytically impossible to order effects and corresponding theories in a cline from “weak” to “strong.” While it may be possible to do so in some cases, within each cell in **Table 2**, one would have to formulate carefully the “metric” for such ordering. Of the four major types of linguistic influence, Types 1, 2, and 4 appear to be both possible, and in some particular cases: *actual*. Hence, they are not mutually exclusive.

## Conclusion

The topic of the relation of language on thought, and in particular the thesis that language influences thought in one or several different ways, is somewhat like that of language origins. First, it has an old pedigree. Second, it fascinates people, and has over the years given rise to many theories, some more plausible than others. Third, it has at times been more or less “banned” due to presumably irresolvable conceptual and methodological problems. In this chapter, we have above all addressed the final point: it is not that anyone has explicitly banned discussion on linguistic influence, in the manner that *La societé de linguistique de Paris* banned papers on language origins in 1886, but there have been persistent attempts to question the viability of the whole research program (Pinker, 1994; Bloom and Keil, 2001; Björk, 2008; McWhorter, 2014).

We have focused on four such attempts, and have argued against them: (1) that it is impossible to disentangle language and thought; (2) that it is impossible to disentangle language from culture and social interaction; (3) that only “trivial” forms of linguistic influence are viable; (4) that all possible forms of linguistic influence can be aligned on a weak-to-strong cline, and the task is to establish which place on the cline is best supported by the evidence. In contrast, we maintained that (1’) it is indeed possible to distinguish language and thought conceptually, since thought (understood as “mediated cognition”) is possible without language; (2’) language is an essential aspect of culture, and is realized through discourse, but this does not invalidate the possibility of cultural influences on thought being separate from language, and vice versa; furthermore, the notion of “language” should be analyzed on several levels and perspectives (see **Table 1**), allowing us to avoid dichotomies like langue/parole, system/discourse or structure/information (3’) the distinction between “trivial” and “interesting” influence stems from a particular view on language and cognition that can be questioned; (4’) at least four different types of linguistic influence can be distinguished, with qualitative differences between them, and that three of these are both feasible and not mutually exclusive.

As they say, the jury is still out on the more empirical claims concerning the influence of language of thought, and our goal has not been to argue in favor of one or another specific mechanism. Rather the aim has been to show that such influence is *possible*, in several different forms. Our hope is that this conclusion, and the conceptual clarifications upon which it rests, may contribute to further careful investigations in order to establish which of these is *actual*.

## Conflict of Interest Statement

The authors declare that the research was conducted in the absence of any commercial or financial relationships that could be construed as a potential conflict of interest.
